# Assessment of MRI Contrast Agent Kinetics via Retro-Orbital Injection in Mice: Comparison with Tail Vein Injection

**DOI:** 10.1371/journal.pone.0129326

**Published:** 2015-06-10

**Authors:** Fang Wang, Masanori Nojima, Yusuke Inoue, Kuni Ohtomo, Shigeru Kiryu

**Affiliations:** 1 Department of Radiology, Institute of Medical Science, University of Tokyo, Tokyo, Japan; 2 Department of Radiology, Qi Lu Hospital of Shandong University, Jinan, China; 3 Division of Advanced Medicine Promotion, The Advanced Clinical Research Center, The Institute of Medical Science, University of Tokyo, Tokyo, Japan; 4 Department of Diagnostic Radiology, Kitasato University School of Medicine, Kanagawa, Japan; 5 Department of Radiology, Graduate School of Medicine, University of Tokyo, Tokyo, Japan; USGS National Wildlife Health Center, UNITED STATES

## Abstract

It is not known whether administration of contrast agent via retro-orbital injection or the tail vein route affects the efficiency of dynamic contrast-enhanced magnetic resonance imaging (MRI). Therefore, we compared the effects of retro-orbital and tail vein injection on the kinetics of the contrast agent used for MRI in mice. The same group of nine healthy female mice received contrast agent via either route. An extracellular contrast agent was infused via the tail vein and retro-orbital vein, in random order. Dynamic contrast-enhanced MRI was performed before and after administering the contrast agent. The contrast effects in the liver, kidney, lung, and myocardium were assessed. The average total times of venous puncture and mounting of the injection system were about 10 and 4 min for the tail vein and retro-orbital route, respectively. For all organs assessed, the maximum contrast ratio occurred 30 s after administration and the time course of the contrast ratio was similar with either routes. For each organ, the contrast ratios correlated strongly; the contrast ratios were similar. The retro-orbital and tail vein routes afforded similar results in terms of the kinetics of the contrast agent. The retro-orbital route can be used as a simple efficient alternative to tail vein injection for dynamic contrast-enhanced MRI of mice.

## Introduction

As animal models are used extensively in biomedical research, small animal imaging has become increasingly important. Affording high spatial resolution and excellent soft tissue contrast, magnetic resonance imaging (MRI) has been applied widely to investigate small animals *in vivo*. As a non-invasive, reproducible technique, MRI is valuable for assessing the longitudinal kinetics of a contrast agent, revealing time-dependent intensity changes within a single animal.

Magnetic resonance (MR) contrast agents can vastly improve image quality, and have made a significant impact on the utility of MRI. In the time since the introduction of contrast agents, the number of contrast-enhanced MRI examinations has increased markedly. Such examinations are widely used for qualitative and quantitative assessments, such as organ-specific imaging [1], dynamic contrast-enhanced MRI [2], and perfusion MRI [3], and so on. For accurate reproducible measurements, dynamic contrast-enhanced MRI requires reliable venous administration of the contrast agent.

The route of injection used most commonly to deliver a contrast agent intravenously in small animals is the tail vein. However, because of the small diameter and fragility of mouse vessels, this procedure remains technically challenging, time-consuming, and requires experience [4]. Repeated injections into the same animal become increasingly difficult, because of vessel stenosis caused by fibrotic changes. Despite concern on potential damage to vision [5], retro-orbital injection is used frequently in many fields; this is as a much easier, faster reproducible technique. After appropriate training, operators can perform this technique reliably. Repeated improper retro-orbital injection may cause possible fibrotic change of surrounding orbital tissues, however, due to easiness, it rarely occurs.

Some studies have compared different routes of injection using different imaging modalities, such as positron emission tomography and bioluminescence imaging [4, 6, 7]. However, it is not known whether the tail vein or retro-orbital administration route affects the efficiency of contrast agent enhancement in dynamic MRI. Therefore, we compared the dynamics of an extracellular MR contrast agent commonly used in clinical practice and preclinical research, after administration via the tail vein or the retro-orbital route in healthy mice.

## Materials and Methods

### Ethics statement

All animal experimental procedures were performed in accordance with the University of Tokyo guidelines for animal care and use, and were approved by the Institute of medical science, the University of Tokyo animal care and use committee (PA10-28).

### Animals

Female 8~10-week-old mice (BALB/c, CLEA Japan, Tokyo, Japan) were used. The mice were maintained under specific pathogen-free (SPF) conditions in an animal facility [8] and given a pelleted regular rodent diet and water *ad libitum*. To reduce signal interference from the gastrointestinal system, the mice were fed autoclaved potatoes 1 day prior to MRI [9].

Anesthesia was induced with 4% isoflurane in air using an isoflurane vaporizer (Murako Medical, Tokyo, Japan), and maintained with 1.5% isoflurane. The mice respired freely during data acquisition. When the respiration rate of the mice was about five per 10 s, they were positioned ventrally in ventral with fore and rear legs outstretched on a polymethylmethacrylate plate using cellophane tape.

### Venous insertion and contrast agent delivery

**Fig 1:**
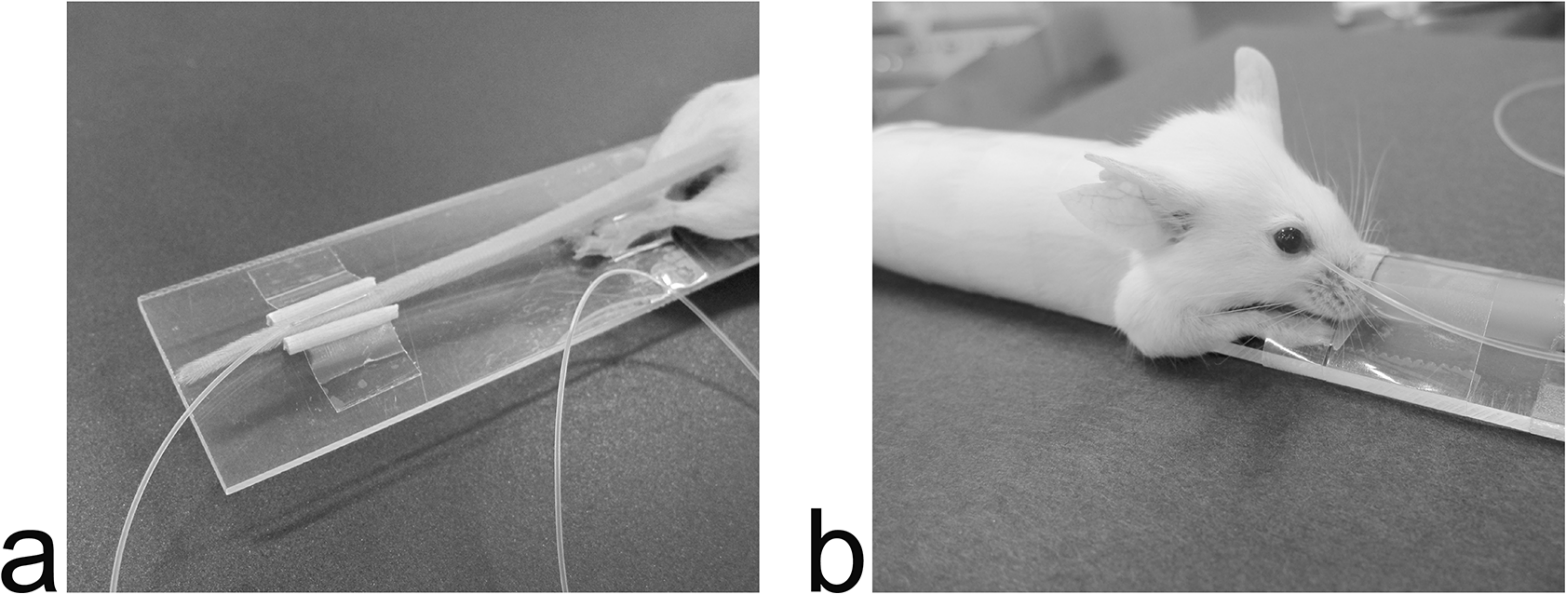
Photographs of tail vein injection and retro-orbital injection. Panel a shows tail vein injection and panel b shows retro-orbital injection in a mouse restrained to the plate holder. A needle-tube system was inserted through the tail vein, while a PE-10 tube was inserted through the medial canthus of the mouse.

For tail vein injection, the self-made needle-tube system, 50 cm long with a dead volume of 30 μL, was used. To build this needle-tube system, we connected a 29-gauge needle cut from a micro-syringe (MYJECTOR; Terumo, Tokyo, Japan) to a polyethylene tube (PE-10; Becton Dickinson, Sparks, MD). The size of the outer diameter of PE 10 is the same as that of a 23-gauge needle, and its inner diameter is the same as that of a 25-gauge needle. The other end of the PE-10 tube was joined to a micro-syringe to allow of injection. To dilate the tail vein, the tail was immersed in water at 40°C for 15 s. The operator stretched the tail, kept the needle as parallel as possible to the tail vein, inserted the ethanol-sanitized needle into the vein without avascularization, and connected the needle with the vein Two cut toothpicks were arranged at each side of the tail as support for the tape and to affix the tail and needle (Fig 1A).

For retro-orbital injection, a PE-10 tube was used (without a metal needle) to avoid severe artifacts caused by the syringe needle during MRI. To render puncture easy, one end of the tube was cut in a beveled manner and the other end was connected to a micro-syringe. The edge of bevel was trimmed to enable smooth insertion. The eye was partially protruded to facilitate PE tubing placement by applying a gentle pressure in the periorbital area. After ethanol-sanitization of tip of the PE-10 tube, with the bevel-down tip position, the operator inserted it into the medial canthus of a mouse at an angle of approximately 30° to the horizontal plane. The tube was introduced so that it followed the edge of the eyeball down, until the tip reached the base of the eye (Fig 1B). The operator put a drop of PBS in the eye to prevent it becoming dry. Once the PE tubing was placed, the operator gently bent it caudalward, with an appropriate radian being necessary to hold the tubing in place, and fixed it along mouse body and tail with tape, and then the other end of the tubing was connected to a micro-syringe.

The total times required for puncture and mounting of the injection systems to the holders were recorded for both the retro-orbital and tail vein approaches. After the needle-tube system or the PE-10 tube was fixed, the mouse was placed in the MRI unit. To acquire arterial phase images immediately after injecting the contrast agent, we left the needle-tube system in the tail vein or the PE-10 tube in the retro-orbital sinus during sequential imaging.

### MRI procedures

MRI was performed using a 1-Tesla permanent magnet compact MRI system (MRmini; MRTechnology, Tsukuba, Japan), consisting of a console and a permanent magnet (Hitachi Metals, Tokyo, Japan), installed in the laboratory of the SPF animal facility. A 30-mm inner-diameter solenoid coil acted as a radiofrequency receiver. The magnet measured 60×64×82 (W×H×D) cm, weighed 1,400 kg, and had an air gap of 10 cm and a magnetic field homogeneity of 3.6 ppm in a 30 mm-diameter spherical volume. A scout image was acquired in the coronal view using a T1-weighted three-dimensional fast low-angle shot (FLASH) sequence, with a TR of 30 ms, a TE of 2.2 ms, a flip angle of 57°, an in-plane matrix of 256×64, eight slab partitions, one excitation, a bandwidth of 100 kHz, an in-plane pixel size 0.26 × 0.52 mm, a slice thickness of 2.08 mm, and an acquisition time of 16 s. After the liver was placed at the center of the field of view, thoracoabdominal coronal images were acquired with T1-weighted three-dimensional FLASH sequence with following parameters: TR 30 ms, TE 2.2 ms, flip angle 57°, in-plane matrix 256 × 64, 16 slab partitions, number of excitations one, bandwidth 100 kHz, and acquisition time 32 s. Using zero interpolation, we reconstructed 32 slices with an in-plane pixel size of 0.26 × 0.52 mm and a slice thickness of 0.52 mm.

### Study protocol

The same group of nine healthy mice was given contrast agent via each venous route. For each mouse, the baseline thoracoabdominal image, defined as the zero time point, was acquired before the contrast agent was injected. Gadopentetate dimeglumine (Magnevist; Bayer Schering Pharma, Osaka, Japan) was injected via the tail vein or retro-orbital route, in random order. A total of 0.2 mL/kg of gadopentetate dimeglumine was diluted with phosphate-buffered saline (PBS) and the injection volume per mouse was fixed at 100 uL. Extravasation of contrast agent into surrounding tissues is absorbed into interstitial spaces quickly, without side-effects. For each mouse, the two injections were separated by at least 24 h to allow adequate clearance of the prior contrast agent. Upon completion of contrast agent injection, a flush of 100 μL PBS was given immediately, to avoid retention of the contrast agent at the injection site. To maintain proper pH level of contrast agent, PBS was used in this study rather than physiological saline. Both the contrast agent injection and the PBS flush lasted 10 s. The time points are referable to the onset of each scan (the beginning of contrast agent injection). For both injection routes, serial MRI images were collected from 30 s to 10 min at 45-s intervals, and 15, 20, 25, and 30 minutes after administering the contrast agent. After the scans were complete, the injection devices were detached and the mice were removed from a polymethylmethacrylate plate. Mice were then returned to their home cages and observed for recovery from anesthesia. Every procedure was performed in the laboratory of the SPF animal facility, therefore medical devices, such as needle, PE-10 tube and micro-syringe were sterilized by gas in advance.

### Data analysis

The time courses of contrast ratios for the liver, kidney, lung, and myocardium were quantitatively assessed on serial MRI images. The signal intensity of each organ before and after contrast injection was measured by manually selecting a hand-drawn region of interest (ROI) using ImageJ (National Institutes of Health, Bethesda, MD, USA). Three ROIs were chosen for the liver, two in the right lobe and one in the left, excluding major vascular structures. For each kidney, one ROI was chosen in the parenchyma of the maximum coronal section, excluding the renal collecting system. One ROI was chosen in the maximum coronal section of each lung field posterior to the heart, excluding major vascular structures. Two ROIs were chosen in the maximum and next-larged coronal section of the heart, with each ROI outlining the contour of the cardiac muscle. The mean signal intensities of all ROIs for each organ were defined as the signal intensities of the corresponding organs. The signal intensities in post-contrast images were divided by those of the corresponding pre-contrast images to give contrast ratios. The time course of the contrast ratio in each organ was assessed.

### Statistical analysis

For the liver, kidney, lung, and myocardium, correlations among the average contrast ratios of the nine mice at different time points, between the retro-orbital and tail vein routes, were determined using the Spearman nonparametric approach. Linear regression was also performed. All statistical analyses were performed using EZR (Saitama Medical Center, Jichi Medical University).

## Results

The average times taken for venous puncture and mounting of the injection system were about 10 and 4 min for the tail vein and retro-orbital routes, respectively. We did not observe severe complication in mice undergoing either retro-orbital injection or tail vein injection. Occasionally, minor complication was observed in some mice. A small drop of blood at injection site was observed for retro-orbital injection and repeated attempts were required due to unsuccessful tail vein injection. Even so, no injury or scarring was observed one week after the injection in the eye or tail. No severe artifacts were seen in MRI images taken using either injection route.

**Fig 2:**
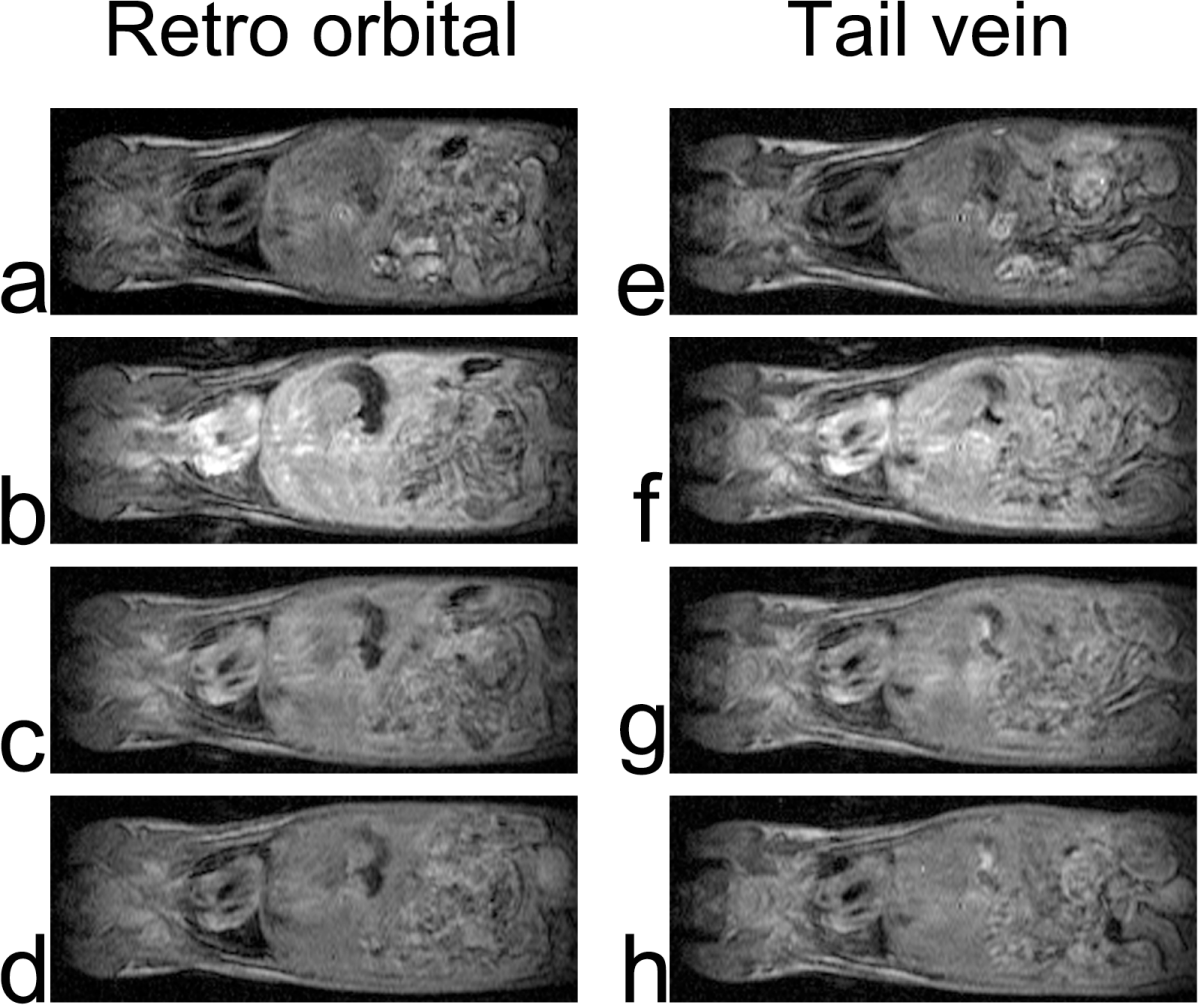
Typical thoracoabdominal coronal MRI. Left column, from top to bottom, images acquired before (a), 30 s (b), 3 min 30 s (c), and 10 min (d) after delivery of the contrast agent via the retro-orbital route. Right column, images obtained before (e), 30 s (f), 3 min 30 s (g), and 10 min (h) after delivery of the contrast agent via the tail vein route.

**Fig 3:**
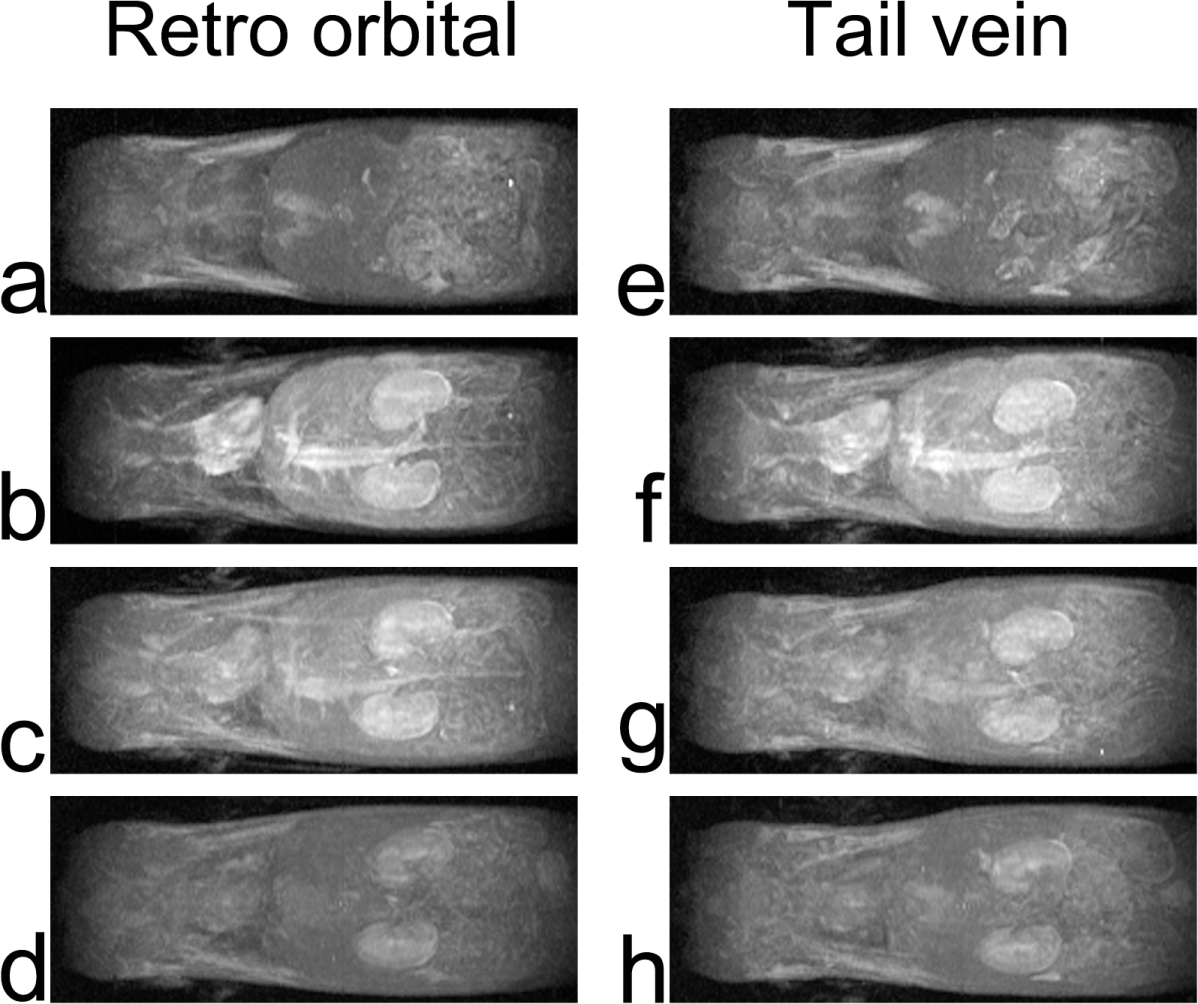
Partial maximum intensity projections of thoracoabdominal MR images. Left column, from top to bottom, images obtained before (a), 30 s (b), 3 min 30 s (c), and 10 min (d) after delivering the contrast agent via the retro-orbital route. Right column, images obtained before (e), 30 s (f), 3 min 30 s (g), and 10 min (h) after delivering the contrast agent via the tail vein route.

Similar levels of enhancement in the liver, kidney, lung, and myocardium were observed using either the retro-orbital or tail vein route. Each organ became enhanced immediately after injection of the contrast agent, and the enhancement decreased gradually (Figs 2 and 3). After 30 min, the kidney showed weak enhancement, and in the other organs, the contrast enhancement had essentially disappeared because of contrast agent wash-out.

**Fig 4:**
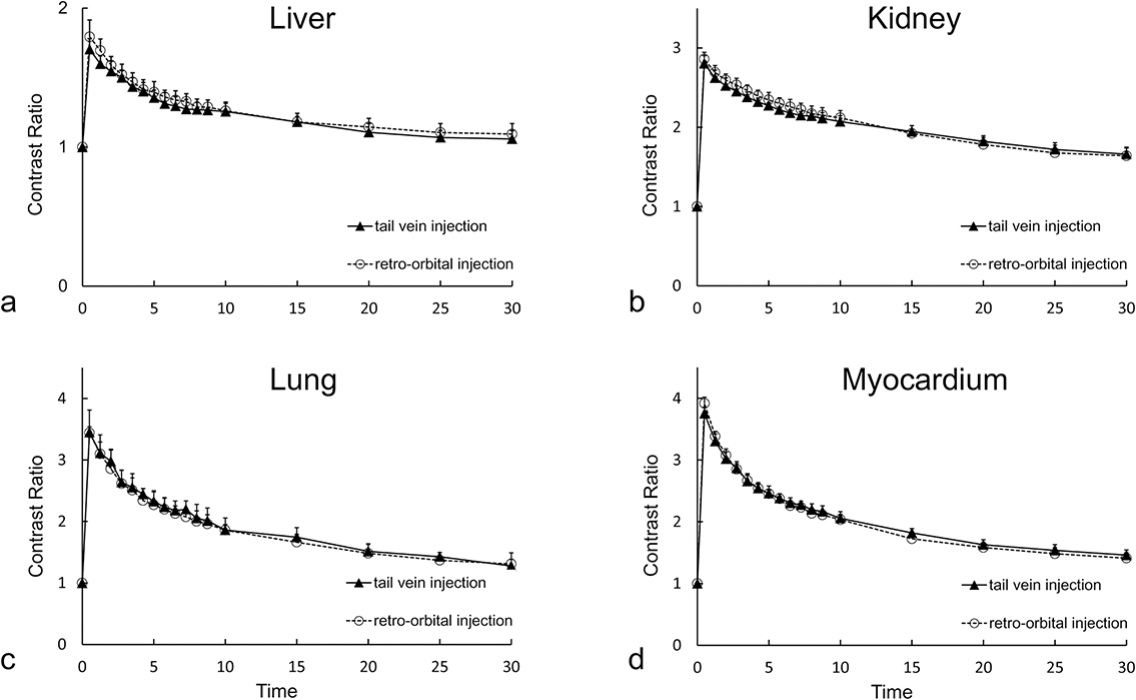
The time courses of contrast ratios after administration via the retro-orbital and tail vein routes. Panel a, b, c and d shows liver, kidney, lung, and myocardium, respectively. Each point represents the mean value of the contrast ratios of nine mice. Time point 0 is before contrast injection and the other time points are from the start of each scan relative to the commencement of contrast agent injection. The error bars indicate standard errors.

The peak contrast ratios in the four organs were obtained 30 s after commencement of injection via either intravenous route (Fig 4). For both the retro-orbital and tail vein routes, the respective peak contrast ratios were 1.79 and 1.70 for the liver, 2.86 and 2.80 for the kidney, 3.44 and 3.45 for the lung, and 3.92 and 3.75 for the myocardium. The contrast ratios for both routes decreased rapidly to 10 min for all organs measured, and then decayed steadily. At 30 min, the contrast ratios for the tail vein and retro-orbital routes were 1.06 and 1.09 for the liver, 1.66 and 1.64 for the kidney, 1.28 and 1.31 for the lung, and 1.46 and 1.41 for the myocardium.

### Statistical results

**Fig 5:**
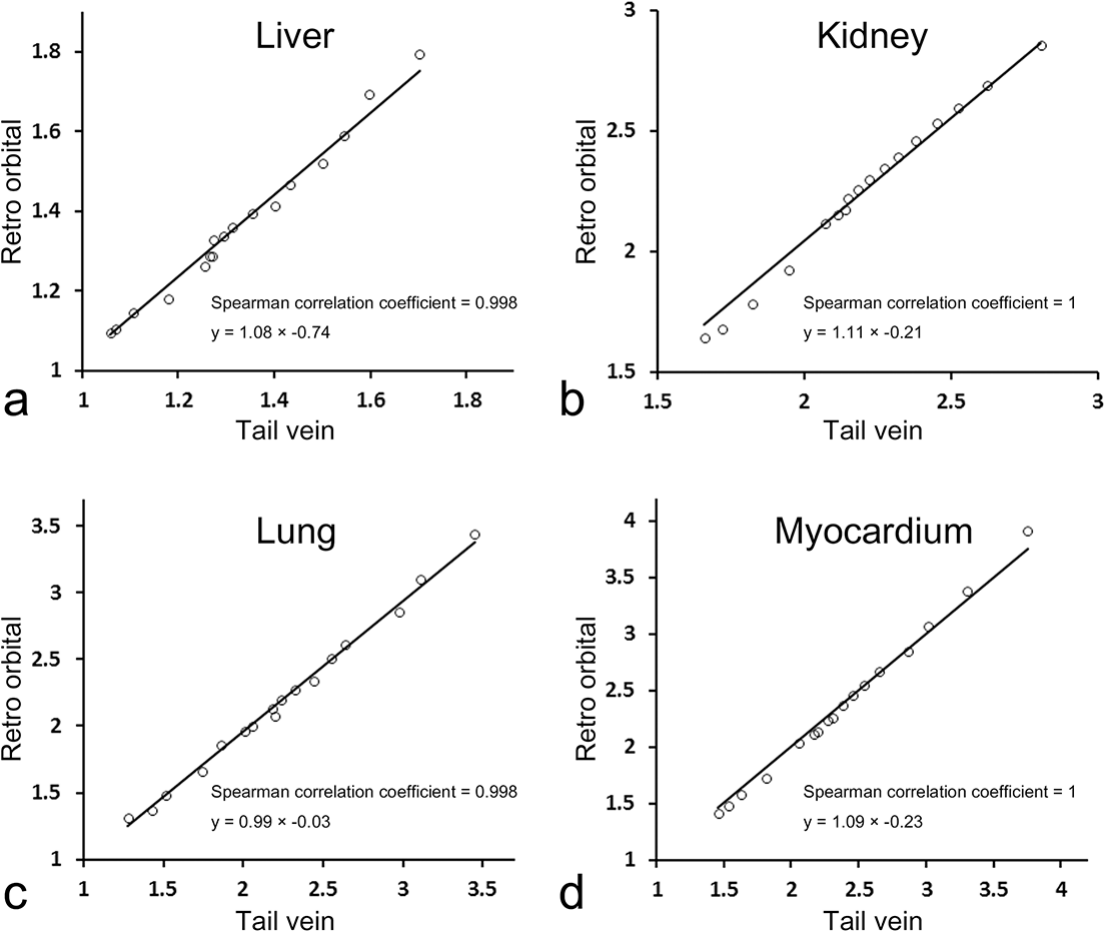
Correlation of contrast ratio between tail vein and retro-orbital route. Panel a, b, c and d shows liver, kidney, lung, and myocardium, respectively.

Significant correlations were evident between contrast ratios obtained using the retro-orbital and tail vein routes. Spearman’s correlation coefficients were 0.998, 1, 0.998, and 1 for the liver, kidney, lung and myocardium, respectively (Fig 5). Upon linear regression, the proportions of contrast ratios between the two routes were close to unity: y = 1.08 × –0.74 for the liver, y = 1.11 × –0.21 for the kidney, y = 0.99 × –0.03 for the lung, and y = 1.09 × –0.23 for the myocardium. Therefore, the contrast ratios were essentially identical when either injection route was used.

## Discussion

We compared the kinetics of an extracellular MR contrast agent after tail vein and retro-orbital injection. For both injection routes, similar enhancement time courses were observed in the liver, kidney, lung, and myocardium. Statistically, the contrast ratios were similar using either injection route in each studied organ at all time points. The average total time required for venous puncture and mounting of the injection system was shorter for the retro-orbital than the tail vein route, and no severe complications were encountered using the retro-orbital route. When the technique is properly done by trained operator, complication was rarely observed. Occasionally, there was a small drop of blood at the injection site, which can be easily wiped with absorbent paper. Consequently, retro-orbital injection is a useful alternative to tail vein injection when dynamic contrast-enhanced MRI is to be performed in mice.

The retro-orbital sinus of the mouse, which is located behind the eye at the medial and lateral canthus, is a confluence of several veins, includes the supraorbital, inferior palpebral, dorsal nasal, and superficial temporal veins [10]. The retro-orbital route is used for blood collection [11], bone marrow engraftment [12], leukemia induction [13], and gene engineering [14]. A study of the delivery of a depletion agent indicated that the retro-orbital route and tail vein routes were similarly effective when macrophage depletion of different tissues was required [5]. Another study concluded that these two routes could be used interchangeably for intravenous injection [15]. A previous study compared the efficiency of retro-orbital and tail vein administration of positron emission tomography radiopharmaceuticals in mice and demonstrated that whereas retro-orbital injection was as effective as tail vein injection, the retro-orbital route was both easier and faster [4]. It has not yet been definitively established whether the retro-orbital route can serve as an alternative to the tail vein route for administration of MRI contrast agents. We found that the enhancement efficiencies of extracellular contrast agents used for dynamic MRI were similar after injection via the retro-orbital and tail vein routes.

The large size of the retro-orbital sinus facilitates injection, saves time, and renders the procedure repeatable [4]. Moreover, the rich capillary microcirculation in the retro-orbital area improves absorption of the injectate, rendering such injection comparable to the tail vein route [4]. For retro-orbital injection, due to the large size of the retro-orbital venous sinus, PE 10 tube can be inserted into a relatively wide space surrounding the eyeball when compared to the tail vein injection. In contrast with the retro-orbital injection, a reliable injection site has to be chosen by operator for the tail vein injection, therefore, it is inevitably time-consuming and injection efficiency is low. In the present study, the average time required for puncture and mounting of the injection system was 4 min for the retro-orbital route, much shorter than the 10 min required for the tail vein route. The first attempt of the retro-orbital injection was almost always successful after appropriate training; however, the tail vein injection is sometimes not successful even in experienced hands.

Any damage caused by retro-orbital injection should be both minimal and transient. Nevertheless, we took care with all manipulations, using PE tubing for injection rather than a syringe, and sterilized devices in advance. We observed no severe complication in mice undergoing retro-orbital injection.

With the PE-10 tube or needle-tube system still placed in the injection site, imaging data were collected immediately after the contrast agent was injected. Thus, the arterial supply to normal or diseased tissue or organs is clearly evident, which sensitively identifies and differentiates pathological conditions.

In the present study, we used a compact MRI system with a 1-Tesla permanent magnet, which has a relatively weaker magnetic field strength than other dedicated MRI systems for small animal imaging. To obtain sufficient signal from a small animal, a superconducting MRI system of high field strength is usually required [16]. However, such MRI system often requires a great deal of space, and their high cost and poor accessibility may preclude the use of MRI in routine experiments. A low-field MRI system can be installed in a small room and requires no special maintenance [16, 17]. With a low field, less chemical shift and fewer susceptibility artifacts are produced [18]. The R1 relaxivity, which determines the T1-shortening effect of a contrast agent, is higher at lower field strengths [19]. In addition, safety issues associated with a static magnetic field are less of a concern at lower field strengths [20].

Upon tail vein injection, the injection needle was left in the vein during the scan, but there was no severe susceptibility artifacts. This may be due to using a low-field magnet. Upon retro-orbital injection, to avoid susceptibility artifacts, a PE-10 tube was used without a needle. The PE-10 tube was both tough and pliable, causing minimal injury to the mouse eye, and was easy to fix after puncture, rendering it suitable for this procedure. We did not observe severe trauma or evidence of infection at the injection site 1 week after injection. Retro-orbital injection via tubing achieved the same effect as tail vein injection with a needle in terms of dynamic contrast-enhanced MRI of the mouse body. In addition, because retro-orbital is free of susceptibility artifacts, this method may be applied to MRI of the mouse head. Moreover, additional contrast agent or drugs can be injected during imaging, which may develop new applications for MR imaging. Different amounts of contrast agent may be tested in a single scan, or the effect of intravenous injection of drug on fMRI can be monitored in real time.

In the present study, we applied three-dimensional FLASH sequence, providing high-resolution images suitable for small animal studies. However, to assess tissue vasculature or tumor neovasculature, much faster imaging sequences are required when a conventional extracellular contrast agent is administered, because of the rapidity of blood circulation in mice. The extracellular contrast agent used in this study was a low-molecular-weight compound that diffuses readily into the extracellular space. The kinetics thereof may differ from those of other MRI contrast agents, such as macromolecular-weight and hepatocyte-specific contrast agents. Therefore, it is necessary to evaluate whether the kinetics of such agents is similar to extracellular contrast agent, given via retro-orbital route.

## Conclusion

The retro-orbital and tail vein routes yielded similar results in terms of the kinetics of MR contrast agents in mice. Retro-orbital injection is a simple efficient alternative to tail vein injection for dynamic contrast-enhanced MRI of the mouse using a conventional extracellular contrast agent.
